# A Novel Frame-Shift Mutation in *SCNN1B* Identified in a Chinese Family Characterized by Early-Onset Hypertension

**DOI:** 10.3389/fcvm.2022.896564

**Published:** 2022-06-14

**Authors:** Yi-Ting Lu, Xin-Chang Liu, Ze-Ming Zhou, Di Zhang, Lin Sun, Ying Zhang, Peng Fan, Lin Zhang, Ya-Xin Liu, Fang Luo, Xian-Liang Zhou

**Affiliations:** ^1^Department of Cardiology, Fuwai Hospital, National Center for Cardiovascular Diseases, Chinese Academy of Medical Sciences and Peking Union Medical College, Beijing, China; ^2^Emergency and Critical Care Center, Fuwai Hospital, National Center for Cardiovascular Diseases, Chinese Academy of Medical Sciences and Peking Union Medical College, Beijing, China

**Keywords:** Liddle syndrome, frame-shift mutation, *SCNN1B*, monogenic hypertension, genetic testing

## Abstract

**Background:**

Liddle syndrome is a form of monogenic hypertension caused by mutations in the three homologous subunits of the epithelial sodium channels (ENaCs), α, β, and γ. It is characterized by early-onset refractory hypertension, hypokalemia, low renin activity, and hypoaldosteronism. In this study, we report a novel frame-shift mutation in *SCNN1B* responsible for Liddle syndrome in a Chinese family.

**Methods:**

DNA samples were collected from all participants. Whole-exome sequencing was performed in the proband to detect possible causative variants. Sanger sequencing was then conducted in the other family members to verify the candidate variant, and in 100 patients with hypertension and 100 normotensive controls to exclude population genetic polymorphism.

**Results:**

We identified a novel frame-shift mutation (c.1691_1693delinsG) in *SCNN1B* that was responsible for Liddle syndrome in this family. This mutation leads to the substitution of Arg in place of Gln at codon site 564 and generates a new stop codon at 592, influencing the crucial PY motif and resulting in reduced inactivation of the ENaCs. Aside from the proband, eight family members carried the mutation. Intra-familial phenotypic heterogeneity was observed in the blood pressure and serum potassium levels. Amiloride therapy combined with a low sodium diet is effective to alleviate the symptoms of patients with Liddle syndrome.

**Conclusion:**

c.1691_1693delinsG, a novel frame-shift mutation in the β subunit of ENaC, was identified in a Chinese family with Liddle syndrome by whole-exome sequencing. Phenotypic heterogeneity can make diagnosis of Liddle syndrome difficult on the basis of clinical or biochemical characteristics alone. Genetic analysis is a useful tool allowing timely and accurate diagnosis of Liddle syndrome and playing a guiding role in precise treatment of the disease.

## Introduction

Systemic hypertension is a commonly recognized health problem affecting over 1.2 billion adults worldwide (1). Chronic elevated blood pressure or hypertensive emergencies can lead to morbid complications such as stroke, heart failure, and renal failure (2). Significantly, 15–20% of cases of hypertension occur secondary to some definable causes, and for those, precise determination of the etiology could help to avoid severe cardiovascular outcomes (3). Monogenic hypertension plays an important role in the development of secondary hypertension, such as Liddle syndrome, apparent mineralocorticoid excess, and congenital adrenal hyperplasia (4).

Liddle syndrome is an autosomal dominant form of hereditary hypertension with early penetrance caused by mutations in the epithelial sodium channels (ENaCs). ENaCs, which are responsible for the rate-limiting step of water and sodium reabsorption in the aldosterone-sensitive-distal nephron, consist of α, β, and γ homologous subunits, encoded by *SCNN1A*, *SCNN1B*, and *SCNN1G*, respectively (5). Any mutation in the subunits can induce the constitutive activation of ENaCs by either accumulative channel density or an increased probability of the channels being open, thereby resulting in excessive sodium reabsorption and volume expansion (6, 7). The disease is classically characterized by severe hypertension, hypokalemia, and metabolic alkalosis in the setting of low/suppressed aldosterone and renin levels (8). However, a variable phenotype of Liddle syndrome is observed in clinical work. Some patients are misdiagnosed and may suffer catastrophic complications because of a lack of awareness of the genotype-phenotype heterogeneity (9). Genetic sequencing, considered a gold standard, is useful and effective for the diagnosis of Liddle syndrome. The blood pressure and potassium levels can be improved greatly by adopting a low potassium diet and tailored therapy including amiloride or triamterene, which are specific antagonists of ENaCs, but patients show no response to spironolactone treatment.

In the present study, we report a Chinese family with Liddle syndrome and identify a novel frame-shift mutation (c.1691_1693delinsG) in *SCNN1B* by whole-exome sequencing. Sanger sequencing indicated that eight family members also carried the variant in addition to the proband. Intra-familial phenotypic heterogeneity was observed in terms of the blood pressure and serum potassium levels. Tailored therapy is beneficial and cost-effective for Liddle syndrome patients to avoid catastrophic complications.

## Materials and Methods

### Subjects

**FIGURE 1:**
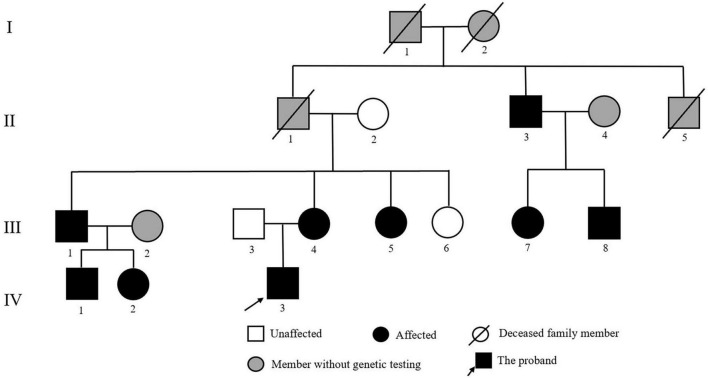
Pedigree of the Chinese family with Liddle syndrome. The arrow indicates the proband; black symbols represent Liddle syndrome patients identified by genetic testing; and gray symbols indicate member without genetic testing.

The proband was an 18-year-old male patient referred to the Department of Cardiology at Fuwai Hospital (Beijing, China) to identify the etiology of his refractory hypertension and hypokalemia. A positive family history of early-onset hypertension was found in his family. In addition to the proband, 11 family relatives participated in this study (Figure 1). Furthermore, to exclude population genetic polymorphism, 100 patients with hypertension and 100 normotensive controls were enrolled into this study, of which the samples’ statistical power was fully assessed by online tool SSizer (10).

### Clinical and Biochemical Characteristics

Imaging examinations, including echocardiography, and abdominal CT of the kidneys, adrenal glands, and renal arteries, were performed on the proband. Laboratory examinations, comprising electrolyte levels, urinary catecholamines, plasma renin activity (PRA), and plasma aldosterone concentration (PAC), were also assessed during the proband’s hospitalization.

### Genetic Testing and Mutation Analysis

We collected venous blood samples from all participants and genomic DNA was extracted using the QIAamp DNA Blood Mini kit (QIAGEN, Hilden, Germany) for genetic testing. Whole-exome sequencing was performed in the proband. After assessing the DNA quality, exomes were captured by Agilent SureSelect Human All Exon V6 kits based on standard protocols and then sequenced using the Illumina HiSeq2000 platform. The raw data were filtered, removing the sequence adaptors and low-quality reads (defined as those with more than 10% undetermined base information in single-end sequencing reads, or low-quality bases in single-end sequencing reads exceeding 50% of the length). Then, the clean reads were mapped to the reference genome (GRCh37/hg19) by Burrows-Wheeler Aligner (beta version^1^), and the Sambamba tool (11) (version: 0.8.2^2^) was used to mark duplicate reads. We used the SAM tool (12, 13) (version: 0.1.19^3^) to identify single nucleotide variants (SNVs)/indels, and the CoNIFER tool (version: 0.3^4^) to detect copy number variation. ANNOVAR software (14) (subversion: 322^5^) was used to annotate filtered variants. *In silico* analysis software, including Polyphen2 (15) (version: 2.2.3), MutationTaster2021 (16), and SIFT,^6^ were used to predict the pathogenicity of variants.

Furthermore, we used Sanger sequencing in other family members to verify the candidate variant. To exclude population genetic polymorphism, we also performed Sanger sequencing on 100 hypertensives and 100 normotensive controls. Polymerase chain reaction (PCR) was used to amplify exon 13 of *SCNN1B* for further familial co-segregation analysis (forward primer, 5′-CCCACCCAAGAATCACCTCC-3′; reverse primer, 5′-TCAGGACAGGTAGGGACGAG-3′) and the results were sequenced by ABI Prism 377 DNA sequencer (Applied Biosystems, Foster City, CA, United States).

### Compliance With Ethical Standards

This study was approved by the Ethics Committee of Fuwai Hospital and was performed in accordance with the Declaration of Helsinki. Informed consent was obtained from every participant.

## Results

### Clinical Characteristics

The proband (IV-3) was diagnosed with hypertension (180/110 mm Hg) at the age of 17 when he went to the hospital complaining of abdominal pain. Since then, he has been treated with anti-hypertensive therapy. However, despite taking amlodipine, spironolactone, and irbesartan hydrochlorothiazide, his blood pressure status was still poor (170/100 mm Hg) and his potassium level was low, fluctuating from 2.38 to 3.45 mmol/L. To identify the etiology of the refractory hypertension with hypokalemia, he was admitted to Fuwai Hospital. Physical examination of the proband, including height, weight, and sexual development, was unremarkable. Biochemical examination indicated hypokalemia (2.67 mmol/L; reference value, 3.5–5.3 mmol/L), low PRA (1.0 μIU/mL; reference value, 2.8–39.9 μIU/mL), low PAC (1.0 ng/dL; reference value, 3.0–23.6 ng/dL), and metabolic alkalosis (HCO_3_^–^, 28.6 mmol/L; reference value, 21.0–27.0 mmol/L; Actual Base Excess, 4.3 mmol/L; reference value, −3.0 to 3.0 mmol/L). Urinary catecholamines and gonadal hormones were within the normal range (Table 1). Microalbuminuria was discovered, and echocardiography showed thickening of the ventricular septum. Results of abdominal CT of the kidneys, adrenal glands, and renal arteries were all normal.

**TABLE 1:** Clinical and biochemical data of the proband during hospitalization.

	Proband IV-3	Normal range
Gender	M	–
Age (years)	18	–
BMI (kg/m^2^)	32.6	18–24
**Blood pressure**		
Highest DBP/SBP (mm Hg)	180/110	140/90
24-h ambulatory DBP/SBP (mm Hg)	150/86	130/80
**Serum electrolytes**		
Potassium (mmol/L)	2.67	3.5–5.3
Sodium (mmol/L)	145.79	137–147
Chloride (mmol/L)	102.38	99–110
**Arterial blood gas analysis**		
pH	7.437	7.350–7.450
pCO_2_ (mm Hg)	43.1	35.0–45.0
PO_2_ (mm Hg)	83.1	80.0–100.0
HCO_3_^–^ (mmol/L)	28.6	21.0–27.0
Actual Base Excess (mmol/L)	4.3	−3.0–3.0
Lac (mmol/L)	1.1	0.5–1.6
sO_2_	97.5%	95.0–99.0%
** Serum hormone concentration**		
Renin, supine/upright (μIU/mL)	1.0/3.8	2.8–39.9/4.4–46.1
Aldosterone, supine/upright (ng/dL)	1.0/1.0	3.0–23.6/3.0–35.3
Aldosterone/renin ratio, supine/upright (ng/dL)/(μIU/mL)	1.000/0.263	<3.7
Testosterone (ng/dL)	245	241–827
Estradiol (pg/mL)	35.0	0–39.8
Cortisol_8*am*_ (μg/dL)	15.7	5.27–22.45
ACTH (pg/ml)	20.1	0–46
**Renal function test**		
Creatinine (μmmol/L)	75.20	44–133
BUN (mmol/L)	3.95	2.86–7.90
URIC (μmmol/L)	426.15	148.8–416.5
mALB (mg/L)	123.9	0–19

*BMI, body mass index; DBP/SBP, diastolic blood pressure/systolic blood pressure; ACTH, adrenocorticotropic hormone; BUN, blood urea nitrogen; URIC, uric acid; mALB, microalbuminuria.*

The proband’s mother (III-4) was diagnosed with hypertension (160/110 mm Hg) at the age of 24 years when she was pregnant. After delivery, her blood pressure slightly decreased, reaching 150/98 mm Hg. His grandfather (II-1) suffered from hypertension when he was 21 years old and died of esophageal cancer at the age of 52 years. In addition, his great-uncle (II-3), maternal uncle (III-1), and elder aunt (III-5) presented with severe early-onset hypertension, and the former (II-3) suffered from a stroke at the age of 60 years old. The proband’s father (III-3), grandmother (II-2), and younger aunt (III-6) were normotensive. Detailed clinical data of family members are listed in Table 2. On the basis of the combination of laboratory results, lack of response to spironolactone and the positive family history, monogenic hypertension was highly suspected for the proband.

**TABLE 2:** Clinical data of family members involved in this study.

Patient number	Gender	Age, years	Age at onset of HT, years	BP, mm Hg	Serum K^+^, mmol/L	Therapy with amiloride after 3 months	
						BP, mm Hg	Serum K^+^, mmol/L
**Affected**							
II-3	M	65	23	160/100	3.32	140/90	3.90
III-1	M	37	25	178/105	2.96	138/85	4.12
III-4	F	41	24	150/98	3.26	140/88	3.87
III-5	F	39	26	155/102	3.17	130/82	3.70
III-7	F	35	34	145/98	3.96	126/86	4.01
III-8	M	39	38	170/106	2.86	136/89	4.17
IV-1	M	10	–	89/60	4.02	–	–
IV-2	F	14	–	92/64	4.15	–	–
IV-3	M	18	17	170/100	2.67	128/80	4.02
**Unaffected**							
II-2	F	67	52	150/93	3.98	–	–
III-3	M	41	–	136/87	4.23	–	–
III-6	F	40	–	134/82	4.15	–	–

### Genetic Analysis

**FIGURE 2:**
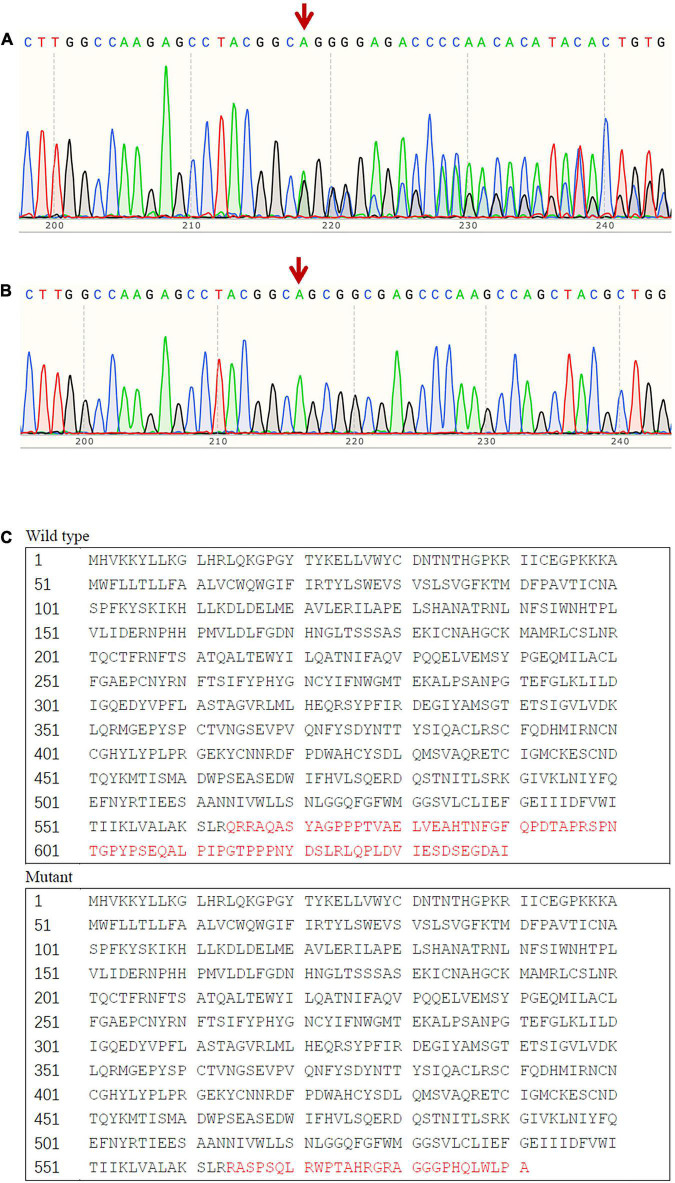
Sequence analysis result of exon 13 of *SCNN1B*. **(A)** The frame-shift mutation (c.1691_1693delinsG) identified in affected patients with Liddle syndrome; **(B)** corresponding normal sequence in unaffected subjects; **(C)** predicted alteration of the protein sequence due to the mutant *SCNN1B*.

We found a novel heterozygous frame-shift mutation (c.1691_1693delinsG) in the *SCNN1B* gene, which was not detected in the gnomAD database, the 1000 Genomes Project database, or the Human Gene Mutation Database. The mutation leads to a substitution of Arg in place of Gln at codon 564 and generates a new stop codon at 592, which influences the PY motif. *In silico* analysis, including MutationTaster2021 suggested that the variant was pathogenic. Sanger sequencing results indicated that there were eight additional family members affected with the causative mutation (Figure 2), but it was not found in any of the 100 hypertension patients or the 100 normotensive controls.

### Tailored Treatment and Follow-Up

All individuals carrying the causative mutation were advised to switch to a healthier lifestyle, including a low sodium diet and more exercise. Additionally, every patient was prescribed a Amiloride/Hydrochlorothiazide combination, each tablet of which contained amiloride (2.5 mg) and hydrochlorothiazide (25 mg), except for IV-1 and IV-2 whose mother refused to accept medicine for them. The patients took one tablet per day, except for the proband and his maternal uncle, who took a double dose of the amiloride because they were overweight. After a 3-month follow-up, the blood pressure and potassium levels improved significantly (Table 2).

## Discussion

In this report, we identified a novel frame-shift mutation (c.1691_1693delinsG) in the *SCNN1B* gene in a Chinese family. Family genetic screening indicated that a total of nine family members were carrying the pathogenic mutation. Intra-familial phenotypic heterogeneity was prominent in this case. Namely, the proband presented with classical Liddle syndrome symptoms, while some members with the same mutation had only mild hypertension and hypokalemia, or even normotension and normokalemia. Tailored therapy, including amiloride and a low sodium diet, play an important role in controlling the disease. Timely genetic testing aids in accurate diagnosis of Liddle syndrome.

In 1963, Liddle syndrome was first described by Liddle et al. in a Caucasian female who presented with hypertension, hypokalemia, and metabolic alkalosis in the absence of hyperaldosteronism (17). In 1994, Shimkets et al. first uncovered the molecular defect of Liddle syndrome by analyzing the β subunit of ENaC in several Liddle syndrome kindreds (18). The disorder results from gain-of-function mutations of *SCNN1A*, *SCNN1B*, and *SCNN1G*, encoding the α, β, and γ subunits of ENaCs, respectively. In the kidney, ENaCs are mainly distributed in the apical membrane of the principal cells located in the aldosterone-sensitive-distal nephron, which play a vital role in maintaining sodium and water reabsorption. Each subunit of ENaC contains a proline-rich segment (PY motif), which is located in the C-terminus and functions by binding the WW domains of the ubiquitin ligase protein Nedd4–2, thereby inducing internalization and proteasomal degradation of the ENaCs. Any deletion or alteration of the PY motif leads to the reduced inactivation of the ENaCs, resulting in an increased density and constitutive activation of ENaCs and consequently followed by excessive sodium reabsorption and plasma volume expansion (19). Additionally, increased sodium reabsorption generates a luminal negative voltage that promotes an elevated secretion level of potassium, leading to abnormal hypokalemia (20).

The worldwide prevalence of Liddle syndrome, irrespective of race or sex, remains unknown. So far, there have been relatively few studies on the prevalence of Liddle syndrome. In 2010, Tapolyai et al. conducted a cross-sectional cohort of 149 patients characterized by hypertension and hypokalemia or metabolic alkalosis and detected a high prevalence (6%, 9/149) of Liddle syndrome biochemical phenotypes (21). In a Chinese early-onset hypertensive population, the prevalence of Liddle syndrome confirmed by genetic testing has been estimated as 1.52% (5/330) and 0.91% (7/766) (22, 23). However, because of the variable penetrance of Liddle syndrome and the incomplete application of genetic testing, the prevalence may be underestimated.

Until now, more than 24 mutations in *SCNN1B* have been reported worldwide, with the most common variants altering the conserved PY motif in the carboxy-terminal cytoplasmic tail of ENaC. The mutation in this report (c.1691_1693delinsG), which is also near the C-terminus, results in the substitution of an Arg amino acid in place of a Gln at position 564 and a truncated open reading frame that ends at position 592. To date, several mutations have been described that are close to the locus reported herein, including missense and nonsense mutations. R563Q has been identified as related to a hypertensive phenotype, and might alter the three-dimensional structure of the β subunit, resulting in weakened intracellular interactions and upregulated ENaC activity (24, 25). Liu et al. identified a nonsense mutation in *SCNN1B* gene (c.C1690T) which introduces a new stop codon instead of a Gln at codon 564 (23). In addition, R566X is considered a hot-spot locus which leads to the removal of the cytoplasmic carboxyl tail (18, 22, 23, 26–31). Previously, Cui et al. screened *SCNN1B* and *SCNN1G* of 12 patients with Liddle syndrome and detected transition from C to T at various sites of *SCNN1B* (c.C1690T, c.C1702T), which leads to premature termination of the protein (28). In renal epithelial cells derived from the distal nephron, Snyder et al. found that βR566X coexpressed with wild-type α and γ subunits generated more than twice as much amiloride-sensitive current as did expression of wild-type αβγ hENaC, indicating that truncation of βhENaC led to increased Na^+^ current (32). A quantitative assay was developed by Firsov et al. (7). This method was based on the binding of a monoclonal antibody directed against a FLAG reporter epitope introduced in the α, β, and γ subunits of ENaC, and demonstrated a significant correlation between the macroscopic amiloride-sensitive sodium current (INa) with the number of ENaC on the plasma membrane (7). Schild et al. investigated sequential deletions in the C-terminus of the βENaC, and reported that a functional domain, – PY motif regulated channel activity (33). Staub et al. found that mutations in the PY motif of βENaC abolished Nedd4-WW binding in the two-hybrid binding assay, and the protein-protein interaction of ENaC with Nedd4 mediated internalization of ENaC (34).

The effect of a frame-shift mutation of *SCNN1B* has been verified in a previous expression study in *Xenopus laevis* oocytes, which is caused by insertion of a cytosine at the codon 594 and truncates the C-terminus at codon 607, disrupting 46 amino acids of βhENaC (32). Snyder et al. introduced serial stop codons into βhENaC and illustrated that deletion of 19 residues was sufficient to reproduce the effect of disease-associated mutations (32). Their data identified that truncation in the C-terminus of the β subunit deleted the conserved PY motif, and induced an increase of transmembranic sodium transport, compared with expression of the wild-type αβγENaC (32). The deletion of the PY motif leads to constitutive activation of the ENaC, and results in Liddle syndrome.

According to previous studies and *in silico* prediction softwares, the frame-shift mutation identified in this report also leads to the absence of the PY motif, by generating a new stop codon at 592 (original stop codon at 641). As a result, the inactivation of the ENaCs is reduced, consequently followed by increased number of ENaC on the plasma membrane and excessive sodium reabsorption. Increased sodium reabsorption leads to plasma volume expansion and promotes secretion of potassium, causing refractory hypertension with hypokalemia of the proband.

Phenotypic variability is common in the clinical and biochemical characteristics of Liddle syndrome, and this was also observed in this study (35, 36). The blood pressure level, degree of hypokalemia, therapeutic effect of amiloride, and targeted organ damage were the key features in the heterogeneity analysis (22, 27). Previously, genotype–phenotype correlation analysis has been conducted in two Liddle syndrome families with various mutations by Gong et al., which revealed the existence of inter- and intra-familial phenotypic variability (27). Even patients in the same family carrying the same pathogenic mutation may present with mild hypertension or even normotension (22). In terms of the serum potassium condition, hypokalemia was observed in 71.8% of Liddle syndrome patients, and 78% of pediatric Liddle syndrome patients (37, 38). In this study, subjects IV-3 and III-1 presented typically with early-onset severe hypertension, whereas subjects III-7 and III-5 showed mild hypertension, and subjects IV-1 and IV-2 had normal blood pressure. Moreover, the incidence rate of hypokalemia in this family was slightly lower than that previously reported, around 66.7%, which may be due to the limited patient number in the pedigree.

Because some Liddle syndrome patients harbor atypical clinical symptoms and biochemical characteristics, they may be easily misdiagnosed with primary aldosteronism or primary hypertension (23, 39). Long-term uncontrolled blood pressure often results in severe disease complications and targeted organ damage, such as heart failure, renal failure, or even sudden death. To avoid these, clinicians should consider Liddle syndrome when they encounter patients presenting with early-onset hypertension, suppressed PRA, low renin level, and hypokalemia. Genetic testing is considered an essential and rapid tool to ensure that patients with Liddle syndrome can be diagnosed in an accurate and timely fashion. In addition, genetic screening of high-risk family members based on the proband is necessary and efficient for detecting potential Liddle syndrome patients. Once diagnosed, ENaC inhibitors (such as amiloride and triamterene) in combination with a low sodium diet should be individualized. Liu et al. reported that an average follow-up study for 4 years of 17 Liddle syndrome patients showed that blood pressure and potassium levels can be controlled well by the adoption of amiloride therapy (23).

## Conclusion

In conclusion, we have identified a novel frame-shift mutation in the β subunit of ENaC in a Chinese family with Liddle syndrome by whole-exome sequencing, further refining the known genetic mutations of *SCNN1B*. Patients with Liddle syndrome may be misdiagnosed when diagnosis is based on clinical or biochemical characteristics alone because of the existence of phenotypic heterogeneity. Genetic analysis is necessary to ensure timely and accurate diagnosis of Liddle syndrome and to apply tailored treatment to prevent the occurrence of disease complications.

## Data Availability Statement

The data presented in the study are deposited in the Sequence Read Archive (SRA) repository, accession number SAMN2886425.

## Ethics Statement

The studies involving human participants were reviewed and approved by the Ethics Committee of Fuwai Hospital. Written informed consent to participate in this study was provided by the participants’ legal guardian/next of kin. Written informed consent was obtained from the individual(s), and minor(s)’ legal guardian/next of kin, for the publication of any potentially identifiable images or data included in this article.

## Author Contributions

Y-TL, X-CL, FL, and X-LZ designed the study and modified the manuscript. Y-TL, X-CL, Z-MZ, DZ, and LS collected the clinical information and performed the data analysis. YZ, PF, LZ, and Y-XL performed the experiments. Y-TL and X-CL wrote the manuscript. All authors reviewed this work.

## Conflict of Interest

The authors declare that the research was conducted in the absence of any commercial or financial relationships that could be construed as a potential conflict of interest.

## Publisher’s Note

All claims expressed in this article are solely those of the authors and do not necessarily represent those of their affiliated organizations, or those of the publisher, the editors and the reviewers. Any product that may be evaluated in this article, or claim that may be made by its manufacturer, is not guaranteed or endorsed by the publisher.

## Footnotes

1
https://sourceforge.net/projects/bio-bwa/

2
https://github.com/biod/sambamba/

3
http://samtools.sourceforge.net

4
http://sv.gersteinlab.org/cnvnator/

5
https://annovar.openbioinformatics.org/en/latest/

6
http://sift-dna.org
